# Glutamine synthetase sequence evolution in the mycobacteria and their use as molecular markers for *Actinobacteria *speciation

**DOI:** 10.1186/1471-2148-9-48

**Published:** 2009-02-26

**Authors:** Don Hayward, Paul D van Helden, Ian JF Wiid

**Affiliations:** 1 DST/NRF Centre for Excellence in Biomedical Tuberculosis Research, US/MRC Centre for Molecular and Cellular Biology, Division of Molecular Biology and Human Genetics, Faculty of Health Sciences – Stellenbosch University, PO Box 19063/Francie van Zijl Drive, TYGERBERG 7505, South Africa

## Abstract

**Background:**

Although the gene encoding for glutamine synthetase (*gln*A) is essential in several organisms, multiple glnA copies have been identified in bacterial genomes such as those of the phylum *Actinobacteria*, notably the mycobacterial species. Intriguingly, previous reports have shown that only one copy (*gln*A1) is essential for growth in *M. tuberculosis*, while the other copies (*gln*A2, *gln*A3 and *gln*A4) are not.

**Results:**

In this report it is shown that the *gln*A1 and *gln*A2 encoded glutamine synthetase sequences were inherited from an *Actinobacteria *ancestor, while the *gln*A4 and *gln*A3 encoded GS sequences were sequentially acquired during *Actinobacteria *speciation. The glutamine synthetase sequences encoded by *gln*A4 and *gln*A3 are undergoing reductive evolution in the mycobacteria, whilst those encoded by *gln*A1 and *gln*A2 are more conserved.

**Conclusion:**

Different selective pressures by the ecological niche that the organisms occupy may influence the sequence evolution of *gln*A1 and *gln*A2 and thereby affecting phylogenies based on the protein sequences they encode. The findings in this report may impact the use of similar sequences as molecular markers, as well as shed some light on the evolution of glutamine synthetase in the mycobacteria.

## Background

Gene duplication is a common occurrence in bacterial genomes and may result from evolutionary pressures exerted on the organism by the niche it occupies, thereby enabling adaptation to changing environments [1-3]. Glutamine synthetases (GS; glutamate ammonia ligase EC 3.6.2) are enzymes present in most living organisms where they are involved in the ATP-dependant synthesis of glutamine from glutamate and ammonium. There are two main GS families, namely GSI, which is further subdivided into a GSIβ and the less common GSIα, and GSII. Both the GSI and GSII enzymes are found in prokaryotes, while the GSI enzyme is largely absent in eukaryotes. Various studies have shown that the genes encoding the various GS sub-types are widely distributed in various organisms and encode proteins that have very conserved catalytic and structurally important regions. This finding suggests that all the GS families diverged from a single ancestral sequence through duplication events prior to the divergence of prokaryotes and eukaryotes [4-7]. The GS sub-classes are distinguishable from each other by specific insertion sequences and mechanisms of regulation [5]. The GSIβ sub-type is subjected to post-translational modification by adenylylation of a conserved tyrosine residue by an adenylyltransferase [8], while GSIα and GSII activity may mainly be regulated through feedback mechanisms. The enzymes also appear to differ in structure; the GS I enzymes form dodecamers [9], while GSII molecules are octamers [10]. The DNA and protein sequences of GS have thus been used as molecular markers in the construction of the phylogenetic relationships between evolutionary diverse prokaryotic and eukaryotic organisms [6,11]. These sequences are considered useful as phylogenetic markers due to their higher degree of sequence variation in comparison with other markers, such as 16S rRNA [12], which are very similar in ecologically related organisms.

Organisms belonging to the phylum *Actinobacteria *have adapted to occupy a wide variety of ecological niches and include species that are major antibiotic producers, as well as various human, animal and plant pathogens. The genome sequence of *M. tuberculosis*, a member of the *Actinobacteria*, revealed that this important human pathogen has four glnA gene copies that may encode GSIβ (*gln*A1 and *gln*A4) and GSII (*gln*A2 and *gln*A3) enzymes [13]. Of the four glnA gene copies, it has been shown that *gln*A1 encodes the main and essential GS in *M. tuberculosis *[14], while the other glnA sequences (*gln*A2, *gln*A3 and *gln*A4) encode functional, but non-essential GS enzymes [15]. Although these glnA sequences have been shown to encode enzymes that catalyse glutamine synthesis, their evolution and importance in *M. tuberculosis *is not well understood. Evidence has been presented that suggests that *M. tuberculosis *GSIβ (encoded by *gln*A1) may have evolved to perform other specialised functions not present in non-tuberculosis causing mycobacteria and may play a role in enabling *M. tuberculosis *to survive during infection and growth in the human host [16,17]. These functions may include the synthesis of poly-L-glutamic acid, a cell wall constituent unique to *M. tuberculosis *that might play a role in maintaining cell wall homeostasis [18].

These observations suggest that *M. tuberculosis *might have been subjected to varying environmental pressures that may have influenced GS sequence evolution. This hypothesis questions the retention of potentially non-essential and/or non-functional sequences in the mycobacterial genome. Furthermore, if such sequences are retained, do they evolve at the same rate as the organism, but with enough changes over time, thereby enabling its use as a marker of evolution? In this report we attempted to study the evolution of the *Actinobacteria*, with specific reference to the *Mycobacteriae*, through a comparison of the GS sequences present in these genomes. The GS sequence data was used to construct *Actinobacteria *phylogenies, which were compared to phylogenies constructed from 16S rRNA and cytidine triphosphate (CTP) synthase genes. Through these comparisons it was determined that the GS sequences may undergo adaptive or reductive evolution due to the different evolutionary pressures exerted by the ecological niche the organism occupies. These differences may lead to subtle differences in phylogenetic reconstructions, although broad phylogenies could be defined.

## Results

### Distribution of glnA sequences in the *Actinobacteria*

**Figure 1:**
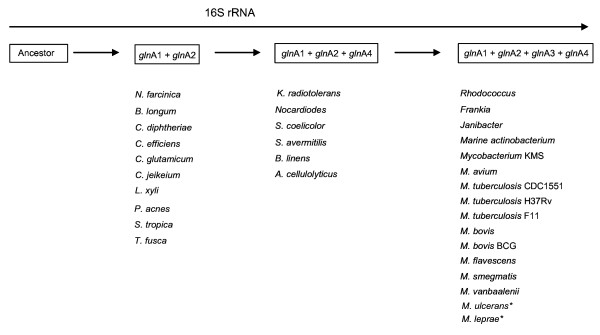
**The distribution of glnA sequences within the genomes of different actinobacterial species reflects the evolutionary history of the phylum *Actinobacteria *as derived from 16S rRNA phylogenetic analyses and indicates that the *gln*A3 and *gln*A4 sequences were acquired in a serial fashion**. *(The *gln*A3 and *gln*A4 sequences are present as pseudogenes in the genomes of *M. leprae *and *M. ulcerans*.)

**Figure 2:**
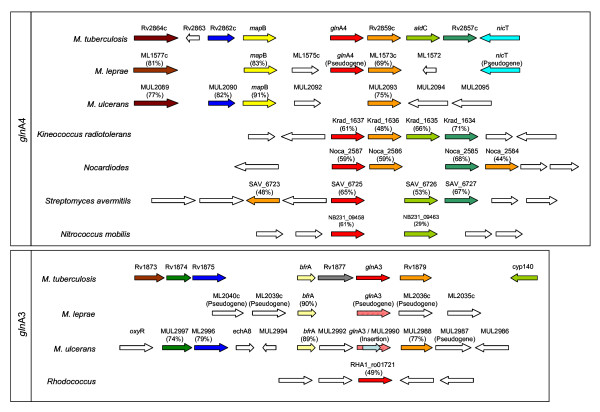
**The chromosomal regions of *M. leprae *and *M. ulcerans *similar to that of *M. tuberculosis *containing the *gln*A3 and *gln*A4 sequences show that these GS encoding sequences were disrupted by insertions (*gln*A3, *M. ulcerans*) or deletions (*gln*A3, *M. leprae*; *gln*A4, *M. ulcerans*)**. Similar genes are indicated in the same colour and the percentage amino acid identity to the *M. tuberculosis *H37Rv reference sequence is indicated between brackets. Open arrows indicate no significant similarity to sequences in the corresponding chromosomal regions.

The distribution and similarity of GS protein sequences in all the available genomes of organisms defined as members of the phylum *Actinobacteria *[19] were detected through a BLAST sequence comparison of the *M. tuberculosis gln*A1, *gln*A2, *gln*A3 and *gln*A4- protein sequences (Table 1). Protein sequence data has been preferred to DNA sequences, since the various *Actinobacteria *genomes may differ with respect to G/C content that may result in skewing of sequence alignments. Protein sequences of high similarity (>60%) to the *M. tuberculosis gln*A1 and *gln*A2 encoded protein sequences could be detected in all the *Actinobacteria *genomes (Table 1), with *Symbiobacterium thermophilum *being the only exception, where only a single GS sequence with greater similarity to the *gln*A1-encoded *M. tuberculosis *GSIβ (50% similarity) was observed. The genome of *S. thermophilum*, a high G+C gram positive organism belonging to an as yet undefined taxon situated just outside the phylum *Actinobacteria*, was included due to its close relationships to the actinobacterial ancestor [19,20]. It was observed that the *gln*A1 and *gln*A2 sequences were situated in close proximity to each other in many genomes, but that considerable variance in the distribution and similarity of GS sequences similar to that *M. tuberculosis gln*A3 and *gln*A4 sequences was observed. Some *Actinobacteria *genomes contained an additional glnA protein sequence similar to the *M. tuberculosis gln*A4 protein sequence. However, this sequence was less conserved than the *gln*A1 and *gln*A2 sequences. Only the mycobacteria and some other closely related actinomycetes, such as *Frankia *and *Rhodococcus *species, contained sequences similar to the four glnA-encoded GS sequences (summarised in Figure 1). An exception was observed in that sequences similar to *gln*A3 and *gln*A4 were absent in the genomes of *M. leprae *and *M. ulcerans*, which had glnA sequences similar to *gln*A1 and *gln*A2 only. It is well known that *M. leprae *and *M. ulcerans *have undergone major reductive evolution [21,22] and as such may have lost these genes. Since the distribution of the glnA sequences (as seen in Figure 1) reflects the evolution of phylum *Actinobacteria *as defined by 16S phylogenetic analysis [19], it might be argued that there was a sequential acquisition of first *gln*A4 and later *gln*A3, rather than a loss of these genes from an actinomycete progenitor. In order to prove that *gln*A3 and *gln*A4 were lost in these two mycobacterial species specifically, rather than being separately acquired in different members of the mycobacteria, the chromosomal regions containing the *gln*A3 and *gln*A4 genes in *M. tuberculosis *were compared to the corresponding chromosomal regions of *M. leprae *and *M. ulcerans *(Figure 2). It was observed that the chromosomal regions of *M. leprae *and *M. ulcerans *contained copies of *gln*A3 in the form of pseudogenes situated in gene clusters corresponding to that of the *M. tuberculosis *H37Rv chromosome. In *M. ulcerans *it was observed that the *gln*A3 sequence had been disrupted by an insertion element (Figure 2). A copy of *gln*A4 can be observed in a gene cluster similar to that found on the *M. tuberculosis *chromosome, suggesting that both sequences have been retained from the mycobacterial ancestor during mycobacterial speciation, but that they have become non-functional through the evolutionary process in some members of the genus *Mycobacterium*.

**Table 1:** GlnA protein sequence distribution and similarity in the *Actinobacteria*

	**Sequence accesion number, length (amino acids) and percentage similarity**							
**Organism**	***gln*A1**		***gln*A2**		***gln*A3**		***gln*A4**	
*Acidothermus cellulolyticus *11B	YP_872682	(474 aa) 72%	YP_872678	(453 aa) 68%	YP_872678	(453 aa) 30%	YP_873609	(446 aa) 61%
*Arthrobacter *sp. FB24	YP_947504	(474 aa) 63%	YP_947491	(446 aa) 65%	YP_831086	(446 aa) 29%	YP_831086	(446 aa) 31%
*Bifidobacterium longum *NCC2705	NP_696248	(478 aa) 62%	NP_696466	(445 aa) 60%	NP_696466	(445 aa) 27%	NP_696466	(445 aa) 29%
*Brevibacterium linens *BL2	ZP_00378605	(474 aa) 62%	ZP_00378066	(452 aa) 62%	ZP_00378066	(452 aa) 29%	ZP_00381218	(454 aa) 56%
*Corynebacterium diphtheriae *NCTC 13129	NP_939986	(478 aa) 67%	NP_940011	(446 aa) 64%	NP_940011	(446 aa) 25%	NP_940011	(466 aa) 28%
*C. efficiens *YS-314	NP_738714	(477 aa) 70%	NP_738737	(516 aa) 66%	NP_738737	(516 aa) 29%	NP_738737	(516 aa) 29%
*C. glutamicum *ATCC 13032	YP_226455	(477 aa) 70%	YP_226471	(446 aa) 65%	YP_226471	(446 aa) 29%	YP_226471	(446 aa) 29%
*C. jeikeium *K411	YP_250482	(500 aa) 71%	YP_250455	(448 aa) 71%	YP_250455	(448 aa) 29%	YP_250455	(448 aa) 29%
*Frankia *sp. EAN1pec	YP_001506114	(474 aa) 66%	YP_001506110	(452 aa) 65%	YP_001510745	(496 aa) 38%	YP_001505022	(470 aa) 56%
*Janibacter *sp. HTCC2649	ZP_00994949	(474 aa) 66%	ZP_00995601 (445 aa) 70%		ZP_00995688 (446 aa) 42%		ZP_00997071	(461 aa) 59%
*Kineococcus radiotolerans *SRS30216	YP_001363019	(474 aa) 68%	YP_001363024	(447 aa) 65%	YP_001363024	(447 aa) 31%	YP_001361387	(460 aa) 61%
*Leifsonia xyli *subsp. xyli str. CTCB07	YP_062980	(474 aa) 62%	YP_061977	(445 aa) 63%	YP_061977	(445 aa) 28%	YP_061977	(445 aa) 32%
*Mycobacterium avium *104	YP_881471	(478 aa) 90%	YP_881448	(446 aa) 94%	YP_882016	(450 aa) 80%	YP_882894	(468 aa) 78%
*M. bovis *AF2122/97	NP_855893	(478 aa) 100%	NP_855895	(446 aa) 100%	NP_855562	(450 aa) 100%	NP_856530	(457 aa) 100%
*M. bovis *BCG str. Pasteur 1173P2	YP_978326	(478 aa) 100%	YP_978328	(446 aa) 100%	YP_978005	(450 aa) 100%	YP_978966	(475 aa) 100%
*M. leprae *TN	NP_301707	(478 aa) 91%	NP_302123	(448 aa) 93%	NP_302123	(448 aa) 27%	NP_302123	(448 aa) 29%
*M. smegmati*s str. MC2 155	YP_888567	(478 aa) 84%	YP_888571	(446 aa) 88%	YP_887864	(453 aa) 64%	YP_886932	(457 aa) 74%
*M*. sp. KMS	YP_939366	(478 aa) 85%	YP_939374	(446 aa) 89%	YP_936250	(437 aa) 47%	YP_938091	(455 aa) 74%
*M. tuberculosis *CDC1551	NP_336749	(478 aa) 00%	NP_336751	(446 aa) 100%	NP_336385	(450 aa) 100%	NP_337439	(457 aa) 100%
*M. tuberculosis *F11	ZP_01685137	(478 aa) 100%	ZP_01685139	(446 aa) 100%	ZP_01684789	(450 aa) 100%	ZP_01685769	(462 aa) 100%
*M. tuberculosis *H37Rv	NP_216736	(478 aa) 100%	NP_216738	(446 aa) 100%	NP_216394	(450 aa) 100%	NP_217376	(457 aa) 100%
*M. ulcerans *Agy99	YP_905364	(478 aa) 90%	YP_905360	(446 aa) 93%	YP_905360	(446 aa) 27%	YP_905360	(446 aa) 30%
*M. gilvum *PYR-GCK	YP_001134193	(478 aa) 84%	YP_001134174	(446 aa) 89%	YP_001134583	(453 aa) 66%	YP_001135323	(469 aa) 74%
*M. vanbaalenii *PYR-1	YP_954385	(478 aa) 85%	YP_954396	(446 aa) 88%	YP_953732	(442 aa) 64%	YP_953098	(459 aa) 72%
*Nocardia farcinica *IFM 10152	YP_117877	(478 aa) 77%	YP_117870	(446 aa) 83%	YP_117870	(446 aa) 28%	YP_117870	(446 aa) 31%
*Nocardioides *sp. JS614	YP_923487	(474 aa) 71%	YP_923242	(455 aa) 66%	YP_923242	(455 aa) 28%	YP_923778	(464 aa) 59%
*Propionibacterium acnes *KPA171202	YP_055385	(473 aa) 66%	YP_055378	(468 aa) 63%	YP_055378	(468 aa) 30%	YP_055378	(468 aa) 30%
*Rhodococcus *sp. RHA1	YP_701142	(478 aa) 81%	YP_701152	(446 aa) 84%	YP_701692	(433 aa) 47%	YP_705251	(451 aa) 33%
*Salinispora tropica *CNB-440	YP_001160144	(474 aa) 70%	YP_001160151	(451 aa) 68%	YP_001160151	(451 aa) 29%	YP_001160151	(451 aa) 30%
*Streptomyces avermitilis *MA-4680	NP_827182	(469 aa) 70%	NP_827131	(453 aa) 69%	NP_827131	(453 aa) 29%	NP_827901	(454 aa) 65%
*S. coelicolor *A3(2)	NP_626450	(469 aa) 71%	NP_626490	(453 aa) 69%	NP_626490	(453 aa) 28%	NP_625889	(462 aa) 64%
*Symbiobacterium thermophilum *IAM 14863	YP_074027	(471 aa) 50%	YP_074027	(471 aa) 33%	YP_074027	(471 aa) 28%	YP_074027	(471 aa) 32%
*Thermobifida fusca *YX	YP_289049	(474 aa) 68%	YP_289043	(453 aa) 68%	YP_289043	(453 aa) 27%	YP_289043	(453 aa) 28%
Marine actinobacterium PHSC20C1	ZP_01131573	(478 aa) 64%	ZP_01129622	(445 aa) 62%	ZP_01129567	(416 aa) 27%	ZP_01129199	(455 aa) 70%

*Gln*A protein sequences distribution in the *Actinobacteria*. The percentage similarity to the *M. tuberculosis *GS sequences is indicated by means of amino acid identity. The accession number and amino acid length of the protein sequence is indicated for each sequence.

### Origins of the *glnA4 *and *glnA3 *sequences

**Figure 3:**
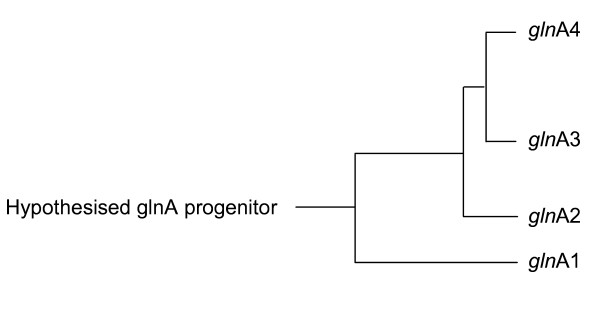
**Phylogenetic analysis of the all the actinobacterial glnA protein sequences showed that the *gln*A3 and *gln*A4 protein sequences are closer related to the *gln*A2 protein sequence that to that of *gln*A1**. (Distances not drawn to scale).

The sequence annotations of the *M. tuberculosis *glnA genes suggest that *gln*A1 and *gln*A3 encode GSI enzymes and *gln*A2 and *gln*A4 GSII enzymes, which together with the results summarised in Figure 1, suggest that the *gln*A4 and *gln*A3 GS sequences were acquired either through sequential duplication of a GSI and GSII sequence, or through separate lateral genetic transfer events. Therefore the ancestry of the glnA sequences was investigated through a phylogenetic analysis of all the glnA sequences present in the phylum *Actinobacteria *(Table 1). The simplified tree shown in Figure 3 (see additional file 1) indicates that, consistent with previous reports, the glnA-encoded protein sequences may have been derived from a common ancestral GS sequence [4]. The sequence phylogeny further shows that the *gln*A2, *gln*A3 and *gln*A4-encoded sequences are clustered on a separate branch from the *gln*A1-encoded sequence, indicating that these sequence are related and may share a common ancestor.

**Figure 4:**
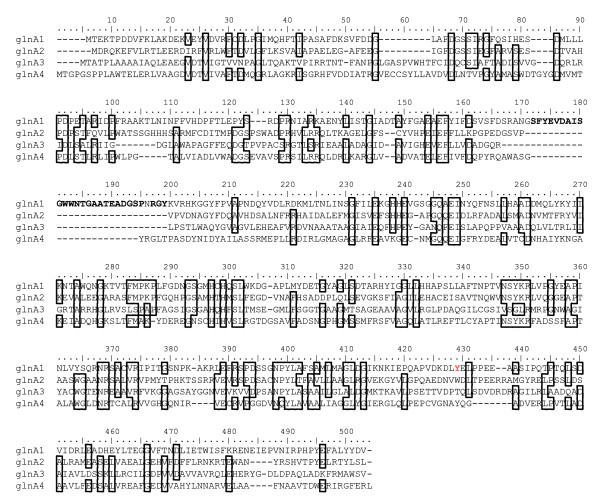
**Multiple protein sequence alignment of the *M. tuberculosis *glnA encoded sequences shows the amount of variation between these proteins**. Identical amino acid sequences are blocked; the insert sequence distinguishing GSIβ are in bold type and the active site tyrosine (position 429) is indicated in red.

This finding was unexpected, since the *gln*A4-encoded GS sequence has a conserved tyrosine residue in the adenylylation region of the GS sequence, suggesting that it may rather be derived from *gln*A1 and would encode a GSIβ enzyme. Therefore the structural relationships between the GS protein sequences encoded by the four *M. tuberculosis *glnA genes were investigated by aligning the *gln*A1 (Rv2220; 478 amino acids), glnA2 (Rv2222; 446 amino acids), *gln*A3 (Rv1878; 450 amino acids) and *gln*A4 (Rv2860c; 457 amino acids) -protein sequences according to maximum probability of amino acid identities (Figure 4). Inspection of the aligned protein sequences of the four *M. tuberculosis gln*A sequences (Figure 4) showed differences in functional regions that separate the GSI and GSII protein families. This data reflects a low level of similarity between the GS sequences due to the low level of sequence conservation in regions containing putative functional domains, notably those that might be involved in the formation of the GS-catalytic site [23]. Furthermore, the protein sequences encoded by *gln*A2, *gln*A3 and *gln*A4 lack the insert sequence that is used to identify GSIβ sequences [5]. In addition, the tyrosine residue in the *gln*A1 protein sequence involved in post-translational regulation of GSIβ through adenylylation [24] is situated in a run of amino acids that is not conserved in the other three proteins. Therefore the tyrosine residue present in the *gln*A4-encoded GS sequence might not be subjected to post-transcriptional regulation by adenylylation, which indicates that the protein sequences encoded by the *gln*A3 and *gln*A4 genes are of the type II GS family. This observation supports the phylogenetic analysis which indicated that the *gln*A3 and *gln*A4 protein sequences are related to or may have been derived from the *gln*A2 protein sequence.

Alignment scores of the GS sequences (calculated as a percentage of amino acid identities per GS sequence length, Table 1) showed that the *gln*A3 and *gln*A4 protein sequences were dissimilar to those encoded by the *gln*A1 and *gln*A2 genes. From the alignment scores it is evident that the protein sequences encoded by *gln*A1 and *gln*A2 are most similar (32.4% – 32.7%, Table 1), while the sequence encoded by *gln*A3 shows the lowest similarity to the protein sequences encoded by *gln*A1, *gln*A2 and *gln*A4 (less than 23%; Table 1). Because it was expected that recent gene duplicates would share a high degree of similarity, the low level of *gln*A4 and *gln*A3 sequence conservation in comparison to the *gln*A1 and *glnA2 *sequences suggests that these sequences either may have undergone rapid evolution after duplication, or have been derived from separate lateral gene transfer events during the speciation of the later actinobacteria. Therefore the *gln*A3 and *gln*A4-encoded protein sequences were compared to all available microbial genomes on the NCBI BLAST server. Sequences with similarity to the *gln*A4 sequence were detected in members of the proteobacteria, such as *Nitrococcus mobilis *(61% similarity) and *Acidiphilum cryptum *(54% similarity). Both these organisms had an additional GSI copy, although it had lower similarity to the *gln*A1-encoded GS of *M. tuberculosis *(50% and 51% similarity respectively). The similarity of these sequences to the *gln*A4 sequence was confirmed by a protein sequence BLAST of the *N. mobilis *protein sequence against all the genomes of the *Actinobacteria*. Higher protein sequence similarity to the *gln*A4 sequence (see Table 1) were observed in all cases, with the sequence of *A. cellulolyticus *(YP_873609) being the most similar (63% identity). In organisms where a *gln*A4 sequence is absent (see Figure 1), no sequences of significant similarity could be detected. However, it could not be conclusively shown whether these sequences were similar enough to suggest that the presence of the *gln*A3 and *gln*A4 sequences could be due to a lateral transfer event. The comparison of the chromosomal regions on which the *gln*A4 gene is found showed remarkable consistency even in more distantly related actinobacteria, while the same was not true for the *gln*A3 gene. For instance, the gene arrangement surrounding the *gln*A4 gene remained the same in *M. tuberculosis *as in *K. radiotolerans*, while very few genes of significant similarity surround the *gln*A3 locus. These observations suggest that the genomic region containing the *gln*A4 gene was inherited from the *Actinobacteria *progenitor, rather than being transferred from an organism outside the phylum. The ancestry of the *gln*A3 gene is more difficult to explain, since a similar sequence could not be detected, suggesting that the *gln*A3 gene arose through a duplication event, but may be undergoing reductive evolution.

### Actinobacteria GS sequences as phylogenetic markers

**Figure 5:**
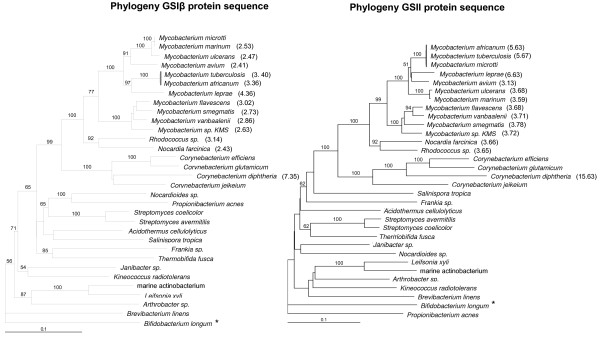
Dendograms of aligned actinobacterial GSIβ (encoded by *gln*A1) and GSII (encoded by *gln*A2) sequences constructed using PAUP 4.0 with the GS sequence of *Bifidobacterium longum *as out-group (*). Percentage bootstrap support values are shown. The ratio of nonsynonymous (K_a_) to synonymous mutations (K_s_) in the GS sequences of the mycobacteria and *C. diphteria *were computed using the GS sequences in *C. efficiens*, and is shown between brackets.

The lower level of GSIβ sequence conservation observed in comparison to the GSII sequence between species (Table 1) was surprising, since GSIβ may be the major GS of *M. tuberculosis *and other *Actinobacteria *[14,15,25]. Since this observation suggests that the GSIβ and GSII sequences evolve differently, *Actinobacteria *phylogenies based on the GSIβ and GSII sequences were compared to phylogenies based on 16S rRNA sequences [19]. Since the *gln*A3 and *gln*A4 protein sequences might be undergoing reductive evolution, they were excluded from the phylogeny. Figure 5 shows that the *Actinobacteria *phylogeny based on the *gln*A2-encoded GSII sequence reflects the 16S rRNA phylogeny, while shifts are observed in the phylogeny based on the *gln*A1-encoded GSIβ sequence. In the GSII sequence phylogeny, organisms are clustered according to suborders, such as the Micrococcineae (*B. linens*, *Arthrobacter*, *L. xyli*, and *Janibacter*), Corynebacterineae (*Corynebacteria *sp., *Mycobacterium *sp., *Rhodococcus *and *N. farcinica*), Streptomycineae (*Streptomyces *sp.), Streptosporangineae (*T. fusca*) and the Frankineae (*A. cellulolyticus*, *Frankia *sp). Exceptions were observed in that *K. radiotolerans *(Frankineae), *P. acnes *and *Nocardiodes *sp. (Propionibacterineae) were dispersed amongst the Micrococcineae. However, bootstrap values below 50 were obtained for these branches making a true interpretation of the inter-relatedness of these organisms impossible. In the phylogenetic tree based on the GSIβ sequence, bootstrap values above 50 were obtained at some of the nodes, but the clustering of organisms to defined *Actinobacteria *suborders were not observed.

The differences in the GS phylogenies are most marked in the mycobacteria. Although the slow-growing and fast-growing mycobacteria are clustered in two separate lineages, only the GSII sequence phylogeny reflects the suggested 16S rRNA phylogeny [26]. For instance, the GSI phylogeny put members of the *M. tuberculosis *complex (*M. tuberculosis*, *M. microtti *and *M. africanum*) in different lineages with *M. ulcerans *and *M. avium *as *M. tuberculosis *complex ancestors. This differs from the GSII phylogeny, which clusters the *M. tuberculosis *complex and puts *M. leprae *and *M. avium *just outside the complex similar to what is observed in 16S rRNA phylogenetic analyses. The branch depth reflects the small amount of variation between the sequences, and the synonymous to nonsynonymous substitution ratio (Figure 5) indicates that there is a selective constraint that preserves the accumulation of amino acid changes over time. However, most of the sequence variation within these sequences occurred outside important functional GS domains. Since phylogenies are not absolute, the results suggest that using GS as a marker in phylogenetic reconstructions gives a broad definition of phylogeny, although subtle differences between trees are observed.

### GSIβ remains conserved between species

Since the sequence encoded by the *gln*A1 locus is the major GS of *M. tuberculosis*, it is expected to undergo little evolutionary change over time. However, the genetic conservation of the gene was studied to assess whether it is subject to gradual changes over time. The *glnA*1 gene (1434 bp) and its 5' and 3' regions were PCR amplified from purified genomic DNA of 54 clinical *M. tuberculosis *isolates. These strains were selected on the basis that they were genotyped by IS*6110 *insertion mapping in a previous study and included highly prevalent and less prevalent strain families as defined in a high tuberculosis incidence community [27]. These clinical isolates are genetically diverse and encompassed the broad *M. tuberculosis *strain families that are grouped according to IS*611*0 banding pattern identities exceeding 65%. The *gln*A1 sequence data obtained in this manner was compared with the corresponding sequences of the *M. tuberculosis *H37Rv reference strain, *M. tuberculosis *CDC1551 and *M. tuberculosis *210 (clinical isolate) through BLAST. The *gln*A1 sequences were 100% similar in all respects and no mutations, deletions or insertions were found in any of the *M. tuberculosis gln*A1 loci, showing that the *gln*A1 sequence undergoes no evolutionary change within *M. tuberculosis*.

## Discussion

Glutamine synthetase has long been considered a good molecular marker for evolutionary studies because, similar to the 16S rRNA gene, it is a universally present and essential component of most living organisms and therefore may be constrained to evolve at a slow rate [4,28]. In addition, the GS sequence is long enough to be used together with other sequences, such as 16S rRNA, to obtain a higher degree of confidence in phylogenetic analyses [29]. However, multiple copies of GS encoding genes have been observed in the genomes of some organisms, notably *M. tuberculosis *(which has four GS encoding genes) [13]. Of these sequences, only the *gln*A1 gene (encoding a GSIβ) has been shown to be essential for *M. tuberculosis *growth, while the other sequences are not [15]. To further understand the evolution of GS and the use of duplicated proteins as evolutionary markers, it was attempted to reconstruct *Actinobacteria *speciation by using GS sequences as phylogenetic markers. Through this study insight was gained into the possible evolutionary scenario of the glnA genes in the mycobacteria.

Through sequence comparisons it was shown that most members of phylum *Actinobacteria *had at least one copy of both the *gln*A1 and *gln*A2 genes and that the protein sequences these genes encode are conserved between species. *Symbiobacterium thermophilum *was an exception having only one *gln*A gene similar to the *gln*A1 sequence. Since *S. thermophilum *may be closely related to the *Actinobacteria *ancestor [19], the absence of the *gln*A2 gene may indicate that *gln*A2 (which is present outside of the phylum *Actinobacteria*) was either not passed down from the *Symbiobacterium *ancestor, or may have been lost from this organism. Previous studies have shown that the GSI and GSII sequences are duplicated derivatives of an ancient GS sequence [4], which suggests that *S. thermophilum *may have lost the *gln*A2 sequence during speciation. It remains to be investigated if other members of the *Symbiobacterium *species may have retained a *gln*A2 gene. It is interesting to note that in many cases, the *gln*A1 and *gln*A2 genes were situated in close proximity to each other. This arrangement has been observed in the genomes of other organisms [30], which suggests that these GS enzymes may be functionally linked. In support of this observation it has been demonstrated that the synthesis of the GSII enzyme was up regulated while the synthesis of GSI was reduced significantly during nitrogen starvation in the *Frankia *[31], therefore suggesting a synergistic role of both enzymes under different conditions. The close proximity of the coding genes for the two GS enzymes also suggests that the chromosomal region containing the glnA copies may be conserved. The genomic region containing the *gln*A2 sequence has been studied in *M. tuberculosis *and *C. glutamicum *and in both cases it was shown that the *gln*A2 gene was situated adjacent to and transcriptionally linked to the *gln*E gene [15,32]. The *gln*E gene encodes the adenylyltransferase involved in the post-translational regulation of GSIβ, and deletion of this gene is fatal owing to disturbances caused from the resulting unchecked GS function [33]. Therefore it is possible that disruptions in the chromosomal region containing the *gln*A2 sequence may be under negative selection pressure.

The distribution and ancestry of the other GS-encoding genes (apart from *gln*A1 and *gln*A2) have not yet been described. The relationships between the *gln*A proteins were investigated by generating a phylogeny of all *Actinobacteria *GS sequences. Through this phylogeny it was revealed that the *gln*A3 and *gln*A4 protein sequences are most closely related to the *gln*A2 protein sequence. Our results suggested that the genes might have been derived from either serial duplications of the *gln*A2 gene, or from separate lateral gene transfer events with *gln*A4 being the first and *gln*A3 the most recent acquisition. Analysis of the functional regions of the GS sequences confirmed the possibility, since it was noted that *gln*A2, *gln*A3 and *gln*A4 encode GSII enzymes. We attempted to establish whether these sequences may have entered the *Actinobacteria *genomes through other mechanisms, such as lateral gene transfer. No clear conclusion could be reached other than that similar sequences were present in some members of the γ-proteobacteria. It is known that lateral gene transfer between mycobacterial species and members of the proteobacteria has occurred [34]. However, these transferred elements are usually related to virulence [35] or pathogenicity [36]. Since GS is involved in central metabolism, no definite conclusion could be made.

The evolutionary history of species within the genus *Mycobacterium *has been investigated using the DNA sequence encoding 16S rRNA [26]. Intriguingly, in comparison to this, subtle differences were observed in the mycobacterial phylogeny based on the GSIβ protein sequence, although the phylogeny based on the GSII sequence reflected the proposed mycobacterial speciation more closely. This observation suggests that, although the coding sequences are constricted as measured by synonymous to non-synonymous substitution rates, change in the GSIβ and GSII sequences may be influenced by environmental pressure. The greater similarity between the GSII sequences may suggest that this sequence remains more conserved and undergoes change at a different rate to the GSIβ sequence. The greater conservation between the GSII sequences indicates that this enzyme might have played a more important role in the early *Actinobacteria *species, although it may have become redundant in some of the later mycobacteria. In this respect, it is interesting to note that deletions of the *gln*A2 sequence lead to attenuation of *M. bovis *in guinea pigs [37], whilst the same result was not observed in mice infected with *M. tuberculosis *strains with *gln*A2 disruptions [38]. From the analysis of actinobacterial genomes containing sequences similar to the glnA sequence, it seems that the *gln*A3 and *gln*A4 duplication event may have occurred independently, since some *Actinobacteria *genomes contain either *gln*A3, *gln*A4 or both, together with the *gln*A1 and *gln*A2 sequences. However, some bacteria, such as *M. leprae *and *M. ulcerans*, might have had a copy of *gln*A3 and *gln*A4, which was lost due to transposon insertions or deletions, suggesting that a lack of *gln*A3, *gln*A4 or both genes might also be due to reductive evolution such as is observed in the genomes of *M. leprae *and *M. ulcerans *[21,39]. If it is accepted that some of the mycobacteria have lost the *gln*A3 and *gln*A4 sequences, this could indicate the redundancy of the GS encoded by these sequences, since if they had a function besides glutamine synthesis they might have been under different evolutionary pressure to be retained in the genome.

The influence of evolutionary pressures on such a critical metabolic enzyme may be explained by adaptive evolution of GS due to pressures exerted by the distinct ecological niches these organisms occupy. Adaptive evolution may lead to functional promiscuity whereby an enzyme can exert other functions, whilst still using the same active site as for the original singular activity [40]. In this respect, it has been shown that the GSIβ enzyme may be exported in great quantities by *M. tuberculosis *and *M. bovis *(also the BCG sub-strains) and that it might be involved in the formation of poly-L-glutamic acid, a cell wall constituent unique to these two mycobacterial species [14]. Evidence has been presented that these functions might be essential for *M. tuberculosis *survival *in vivo *[18], and that the GSIβ enzyme may have functions that contribute to the virulence of these important human pathogens, which cannot be substituted by the GSIβ from non-pathogenic mycobacteria (such as *M. smegmatis*) [38]. The ability of the GSI sequence to undergo evolutionary specialisation may be the underlying reason why this enzyme has been functionally replaced by the more evolutionary stable GSII sequence in eukaryotes. It was suggested that the GSII enzyme is present in eukaryotes due to lateral transfer from endosymbionts early in the eukaryote evolution and, that in some cases, these eukaryotes had other GS-enzymes that were functionally replaced by GSII [41]. Indeed, a remnant of GSI, lengsin, has been observed in the vertebrate eye lens [42,43]. Lengsin has a dodecameric structure and conserved GSI functionally important regions, but is not catalytically active and has undergone significant evolutionary change in the N-terminal region and probably specialised to play a role in lens homeostasis and transparency.

## Conclusion

In conclusion, the specialisation of critical metabolic enzymes may have implications for the use of such enzymes as molecular markers for evolution. Although diversity in these protein sequences may be useful for discriminating between closely related species that show little variance in the 16S rRNA sequences [28], adaptive evolution of these sequences may skew phylogenies.

## Methods

### Sequence retrieval and multiple sequence alignments

*Mycobacterium tuberculosis gln*A1, *gln*A2, *gln*A3 and *gln*A4 protein sequences were retrieved from Genolist (Pasteur Institute) [44] and compared to the *Actinobacteria *genome databases on the NCBI microbial genomes BLAST server [45]. Glutamine synthetase protein sequences were retrieved and compared through multiple sequence alignment using ClustalW 1.8 software at the European Bioinformatics Institute [44,46]. The alignments were manually checked for errors using BioEdit 5.0.9 [47]. For phylogenetic reconstructions, some alignments were manually edited during which unaligned regions (inserts) were removed. BLAST searches against the genomes of *M. africanum*, *M. marinum *and *M. microtti *were carried out on the Sanger Institute website [48] by using the function TBLASTN.

### Phylogenetic trees

The edited GS protein sequences were subjected to phylogenetic analysis using the neighbour joining algorithm (PAUP 4.0*; Phylogenetic Analysis Using Parsimony (*Other Methods) Version 4b10. Sinauer Associates, Sunderland, Massachusetts). A 1000 subsets were generated for bootstrap resampling of the data to establish a degree of statistical support for nodes within each phylogenetic reconstruction [49]. A consensus tree was generated using the program contree (PAUP 4.0*) in combination with the majority rule formula. The GS protein sequence of *Symbiobacterium thermophylum *was selected as out-group to assign roots due the closer relation of this organism to the *Actinobacteria *ancestor [19]. Only branches which occurred in > 50% of the bootstrap trees were included in the final tree and all branches with a zero branch length were collapsed. Overall topology of the trees were confirmed using PhyML 3.0 [50] (data not shown). Synonymous (K_s_) and non-synonymous (K_a_) substitutions were calculated using DnaSP software [51]. In these calculations, the *gln*A1 or *gln*A2 DNA sequence of *C. efficiens *was selected as the out-group.

### *M. tuberculosis *clinical isolate DNA preparation and *glnA1 *sequencing

DNA was isolated from *M. tuberculosis *clinical isolates representative of the various strain families [52] and genotypically classified through the internationally standardised IS-3' fingerprinting method [53]. The Southern-blot autoradiographs were normalised and the IS-3' bands were assigned using GelCompar software (version 4.1). Assignments were visually checked by two independent persons and bands with a >20% intensity than the other bands were scored as representing the IS*6110*-mediated evolutionary events [54]. This DNA was used as template for the PCR amplification of glnA1 using the primers listed in Table 2. PCR reactions were carried out in a GeneAmp 2500 PCR-system (Perkin Elmer) with an initial enzyme activation and DNA denaturing step of 15 min 92°C, followed by 30 cycles at 92°C (2 min); T_m _(Table 3, 30 sec) and 72°C (1 min) and a final 7 min elongation step at 72°C. PCR products were purified using the Promega SV-miniprep system and submitted for direct automated DNA sequencing (Central Analytical Facility, Stellenbosch University, South Africa). Full-length *gln*A1 sequences were assembled from sequencing data using DnaMan software and compared to each other through multiple sequence alignment using ClustalW 1.8 software [44].

**Table 2:** GlnA protein sequence similarity in *M. tuberculosis*

	*gln*A1	*gln*A2	*gln*A3	*gln*A4
*gln*A1	-------	32.5	17.1	22.3
*gln*A2	32.5	------	19.9	30.2
*gln*A3	17.1	19.9	------	24.3
*gln*A4	22.3	30.2	24.3	------

Alignment similarities of the *M. tuberculosis gln*A2, *gln*A3 and *gln*A4 protein sequences to each other showed that these sequences are largely unrelated.

**Table 3:** PCR primer sequences and priming sites

**Name**	**Sequence (5'-3')**	**Product size:**	**Pair Tm (°C)**	**Genome Coordinates**
glnA Up F	AGATGGACACGGTGGAGT	796 bp	55	2486860
glnA Up R	CTTTACTGTATCCGCGGC			2487605
AI FI	CACGGTCAGTAACGTCTGC	550 bp	55	2487524
AI RI	TCCACCTCGTAGAAGGAGC			2488081
AI FII	TTCGATTCGGTGAGCTTC	574 bp	57	2488029
AI RII	GCCGCTTGTAGGAGTTCA			2488602
AI FIII	ACGACGAGACGGGTTATG	294 bp	54	2488483
AI RIII	ATCAGCATGGCCGAGAAC			2488768
AI FIV	TGGTCTATAGCCAGCgcA	597 bp	56	2488633
AI RIV	GAGATGATTGCCAAGCGG			2489229

Polymerase chain reaction primers used to amplify the *gln*A1-locus of *M. tuberculosis*, including its' 5'- and 3' surrounding regions, as overlapping PCR fragments, which facilitated the assembly of the full target region for sequencing (2369 bp).

## Authors' contributions

DH carried out all experimental work, interpretation of data and drafted the manuscript. PvH and IJFW were responsible for initiating the project and revising the manuscript for intellectual content.

## Supplementary Material

Additional file 1 ***Actinobacteria *phylogenetic reconstruction based on glnA protein sequences.** The data provided represent the phylogeny of several Actinobacteria based on the glnA protein sequences present in these genomes. Click here for file
