# Supplementary material for: Glutamine synthetase sequence evolution in the mycobacteria and their use as molecular markers for Actinobacteria speciation
Source: BMC Evol Biol. 2009 Feb 26;9:48. doi: 10.1186/1471-2148-9-48 (PMC2667176; doi:10.1186/1471-2148-9-48)
Supplement: Additional file 1 — Actinobacteria phylogenetic reconstruction based on glnA protein sequences. The data provided represent the phylogeny of several Actinobacteria based on the glnA protein sequences present in these genomes. [file 1471-2148-9-48-S1.pdf]

Hayward *et al*. 2008

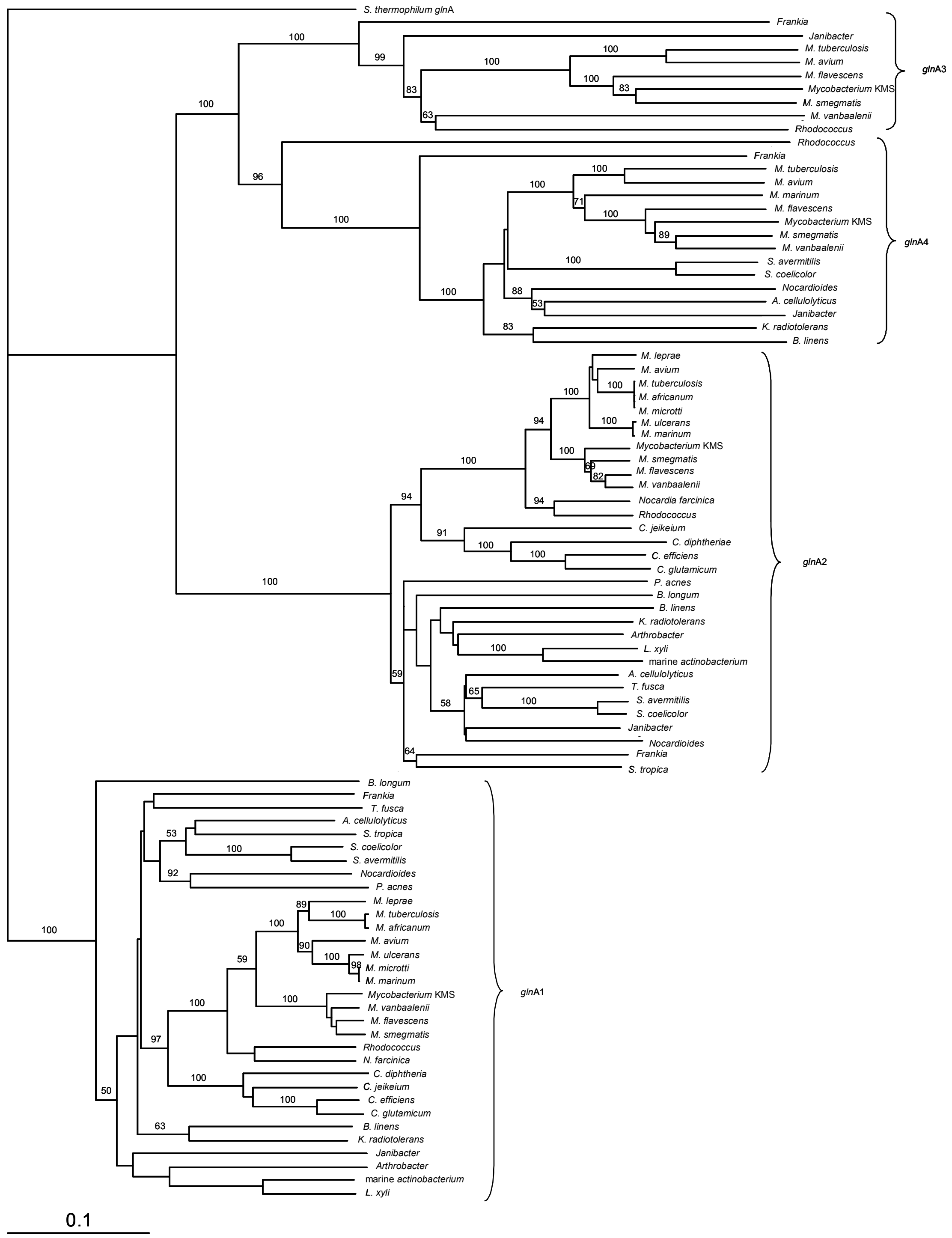

Supplementary Figure 1: A phylogeny of all *Actinobacteria* glnA protein sequence homologs were constructed using the glnA sequence of *Symbiobacterium thermophilum* as out-group. This reconstruction highlights the clustering and evolutionary relationships of the *gln*A1, *gln*A2, *gln*A3 and *gln*A4 sequences, and shows that the *gln*A3 and *gln*A4 sequences are closely related and in turn more related to the *gln*A2 sequence than the *gln*A1 sequence. Percentage bootstrap values are shown.
